# The Impact of Selected Laser-Marking Parameters and Surface Conditions on White Polypropylene Moldings

**DOI:** 10.3390/polym14091879

**Published:** 2022-05-04

**Authors:** Piotr Czyżewski, Dariusz Sykutera, Mateusz Rojewski

**Affiliations:** Department of Manufacturing Techniques, Bydgoszcz University of Science and Technology, Al. Prof. S. Kaliskiego 7, 85-796 Bydgoszcz, Poland; sykutera@pbs.edu.pl (D.S.); mateusz.rojewski@pbs.edu.pl (M.R.)

**Keywords:** laser marking, polypropylene, laser-marking additives LMA, color measurement, surface finishing, topography analysis

## Abstract

Laser marking of polymer materials is a technology that is increasingly used in industry. Polypropylene (PP) shows a low ability to absorb electromagnetic radiation in the near-infrared range (λ = 1064 nm). The paper presents the influence of the surface condition of white-colored polypropylene moldings on the efficiency of their marking with a laser beam. In addition, the operation of the commercial laser marking additive (LMA) Lifolas M 117009 UN, intended to support the process of laser marking of polyolefin surfaces, was verified. The study is an attempt to combine laser operating parameters, material, and geometric properties of PP moldings to obtain the expected quality of graphic symbols. The test samples were made by injection molding method with the use of a specially designed modular injection mold. The molding cavities were prepared with various methods of metal processing, thanks to which obtained moldings differed in surface condition. The marking effects were assessed based on colorimetric tests and digital image analysis. The 0.5 wt% LMA content resulted in obtaining a graphic sign with high contrast in comparison to the background. The gradual increase in the modifier content resulted in a further increase in contrast. These values depended on the degree of surface finish of the samples, and therefore on the roughness parameters. Samples with a rough surface finish showed higher contrast compared to surfaces with a high surface finish. It was also found that for the analyzed moldings, the laser-marking process should be performed with the use of a low head velocity (450–750 mm/s) and a high concentration of the laser beam (0.03–0.05 mm).

## 1. Introduction

Polypropylene (PP) is the most commonly used thermoplastic material in industry. Chemical resistance, easy processing, and low cost are just some of the advantages that speak for using PP in different sectors of the economy (including automotive, packaging, and household equipment sectors). One of the main disadvantages of PP is the high value of processing shrinkage, resulting from the formation of the crystalline phase during cooling [1]. It can be limited, for example, by using mineral or fibrous fillers [2,3,4] and manufacturing process modification by a blowing agent [5]. To provide the products with excellent physical properties and appearance, some of them are further processed by modifying the surface layer. In the age of Industry 4.0, a great emphasis is placed on precise monitoring and production management. This requires graphic symbols such as production date, expiry date, bar code, or serial number be applied to surfaces of the products [6]. The traditional methods used so far, such as screen printing, pad printing, or inkjet printing, are ultimately more expensive, less accurate, and more difficult to remove during recycling processes. Therefore, in the current circular economy, these methods are replaced by technology that uses a beam of laser radiation to apply graphic signs [7]. Laser marking allows for the quick and precise marking of products as well as their efficient identification. The markings made in this way are insoluble in water and have much higher abrasion resistance than traditional markings [8]. Compared to inkjet printers, laser-marking devices do not use inks and chemical reagents, which makes them much more environmentally friendly. The major disadvantages of this method include the high investment cost, the inability to mark in many colors, and knowledge required about the properties of marked materials [9]. In recent years, laser-marking technology has found its application in many industries for marking products made of various materials such as metals [10,11], polymeric materials [12,13], and ceramics [14].

During laser marking, many physical phenomena occur simultaneously on the surface of a sample. Electromagnetic radiation is absorbed by the polymer matrix and then converted into thermal energy. The main observed effect is the process of carbonization of the material in the skin layer, during which the energy of the laser beam locally increases the temperature, causing thermal degradation of the polymer. The presence of oxygen during the process is a source of black or dark contrast, the shade of which depends on the amount of absorbed energy [15]. The second main cause of the observed surface effects is the foaming process, during which the laser energy is absorbed due to the additives contained in the polymer matrix. The supplied heat causes the blowing agent to chemically decompose, releasing water vapor, which is trapped under a thin surface layer of the polymeric matrix [16]. As a result, the graphic sign is the effect of changes in the surface condition due to the local influence of a high-energy beam.

The reaction of the sample surface to the laser beam energy is largely dependent on the properties of the material and the wavelength of the laser radiation (laser radiation absorption coefficient). Neodymium-doped yttrium aluminum garnet (Nd: YAG) lasers with a wavelength of 1064 nm are the most commonly used devices for the modification of plastics [17]. Some of the polymers are susceptible to laser light at this wavelength, creating carbonized dark marks on their surface. These include, among others, polycarbonate (PC) [18], polystyrene (PS) [19], and polyethylene terephthalate (PET) [20]. On the other hand, popular materials from the group of polyolefins, such as polyethylene (PE) or polypropylene (PP), do not absorb laser energy in the range of λ = 1064 nm, creating a blurred graphic symbol [21,22]. The marking efficiency depends to the greatest extent on the degree of surface finish before the process and on the presence in the polymer matrix of laser-sensitive additives from the LMA group (Laser-Marking Additives). It is common practice to use two or more additives. Some are aimed at increasing the absorption of laser radiation, while others improve the optical properties of the surface layer [23].

Commercially available laser-marking additives can provide high marking contrast and edge accuracy. In the case of PP, scientific studies are carried out in two ways. The matrix is modified with pre-made LMA additives, or special powders are prepared. Those additives have to be easy to use, and they must not decrease the physical properties of the product [24]. Carbon nanotubes and graphene, which are coated with a polycarbonate, are used as additives. Liu. et al. [25] also showed that adding Sb_2_O_3_ or Sb_2_O_3_-g-PS to the PP matrix at a concentration of 2.0 wt% results in dark graphic signs on the white-colored surface of their material. Cheng et al. proved that the polypropylene samples filled with ATO@PI powder (antimony-doped tin oxide was coated with polyimide) with the “core-shell” structure can be marked using a laser beam. In this case, the carbonization process is predominant [26]. Using the optical properties of graphene, Wen et al. [27] showed that the addition of 50 ppm of graphene to the PP matrix results in obtaining dark marks on white samples’ surfaces under the influence of laser radiation with a wavelength of 1064 nm. However, articles in which authors combined the laser-marking effectiveness of white-colored moldings with the roughness of the marked surface were not found. Yang et al. have studied the influences of carbon nanotubes/polycarbonate (CNTs/PC) on the photothermal conversion effect during laser marking of a modified polypropylene. It has been proven that the addition of CNTs/PC powder effectively enhances the contrast between the pattern obtained with the laser marking and the surface of the PP sample [28].

Based on the scientific literature, it can be seen that laser-marking tests are carried out on thin-walled PP and PE samples obtained by the pressing method. The prepared LMA powder, usually in amounts up to 200 ppm, is mixed with a polypropylene matrix, which is heated in a mold and pressed [29]. The method of ensuring greater homogeneity of the material is the injection-molding process. The blending process takes place in a plasticizing unit and is more repeatable. In addition, this method gives the opportunity to better shape the surface layer of moldings, primarily through the temperature settings of the mold and the polymer melt. In this way, the amount of crystalline phase in the PP and the surface state of the molded parts can be controlled. Czyżewski et al. have found that roughness is of significant importance during laser marking of black-colored polypropylene moldings [30]. It can be added that the laser-marking tests on the moldings are of great functional importance, as this manufacturing process (injection molding) is one of two most important in the processing of polymers.

The research aimed to assess the influence of the surface state of injection moldings made of white-colored polypropylene on the level of contrast between the laser-applied graphic pattern and the background. An additional goal was to determine the influence of the LMA content in the PP matrix and laser operating parameters on the accuracy of the graphic pattern and its contrast to the background. The marking effectiveness was assessed based on surface topography analysis and colorimetric tests.

## 2. Materials and Methods

### 2.1. Materials

For preparation of samples, homopolypropylene with the trade name Moplen HP 500N (Basell Orlen Polyolefins, Płock, Poland) was used. The selected material is intended for injection molding. The material has the following properties: mass flow rate of 12 g/10 min (230 °C/2.16 kg), the tensile modulus of 1550 MPa, and the tensile strength of 35 MPa. In addition, Weiss K 70 color masterbatch (Lifocolor Farben GmbH & Co. KG, Lichtenfels, Germany) and the laser-marking additive named Lifolas M 117,009 UN (Lifocolor Farben GmbH & Co. KG, Lichtenfels, Germany) were added to the polymer.

### 2.2. Samples Preparation

Four types of PP samples were prepared. They contained 2 wt% color masterbatch and different amounts of LMA. The LMA content in individual compositions was 0 wt%; 0.5 wt%; 1.5 wt%; and 2.5 wt%, respectively. The choice of the LMA used was determined by its dedication to polypropylene applications and economic analysis. Compounds were plasticized in the W25-30D single-screw extruder (Metalchem Plastics Processing Institute, Toruń, Poland). The screw diameter (D) was 25 mm, and the ratio of the screw length to its diameter (L/D) was 30. In the dosing zone, the screw had elements that intensified the mixing. The processing parameters are presented in Table 1. The extruded filament was granulated on a cold granulation line. The homogenized materials with additives were dried in the FED 115 climate chamber (Binder GmbH, Tuttlingen, Germany) for 24 h at a temperature of 110 °C.

**Figure 1:**
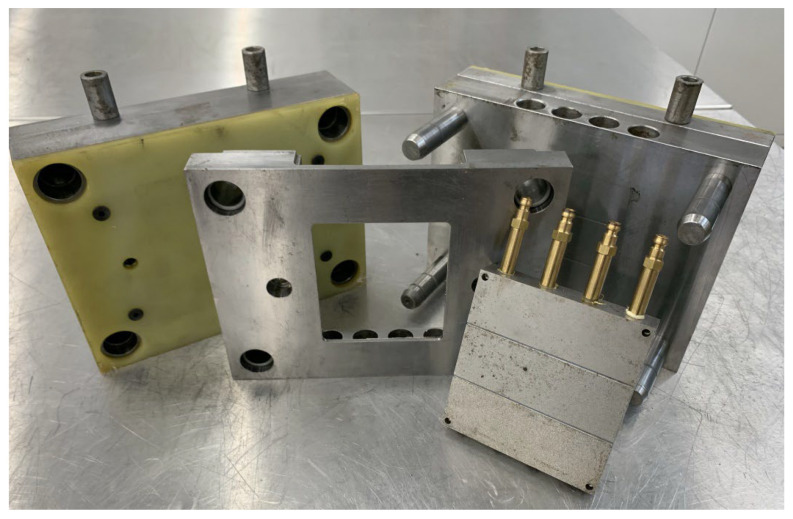
View of a modular injection mold with a forming insert.

Due to the aim of the paper, test samples were made by injection molding with the use of a Battenfeld Plus 350/75 machine (Battenfeld Kunststoffmaschinen GmbH, Kottingbrunn, Austria). A specially prepared modular injection mold was used to manufacture moldings. The modularity of this design included the ability to efficiently change the steel forming insert, which was divided into three fields with the same surface area but different degrees of finishing (Figure 1). Samples with dimensions of 108 × 94 × 2 mm^2^ were tested. The obtained samples were characterized with different surface conditions. The first group of moldings was obtained with the use of forming insert prepared by polishing (marking P), grinding (middle part of the insert—marking S), and honing (marking O). The second mold cavity was produced using electrical discharge machining (EDM) with three different surface finish parameters (designated as DA, DB—middle part, and DC). Therefore, the second group of samples was characterized by greater roughness. The molten material, after being injected under high pressure into the injection mold, reproduced the surface of the cavity. All parameters of the injection molding process are presented in Table 2. After a change in the LMA content, the plasticizing unit was cleaned with the use of PP Moplen HP 500 N material (Basell Orlen Polyolefins, Płock, Poland).

### 2.3. Laser Marking

**Figure 2:**
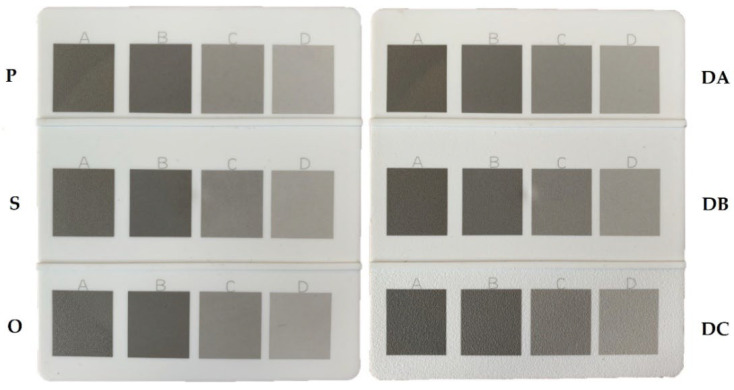
Effect of marking the surfaces of samples with indications of the adopted laser parameters (A, B, C, D—explanation of the marking in Table 3). Molding surfaces: polished (P), ground (S), honed (O), EDM (DA), EDM (DB), and EDM (DC).

The obtained samples with different LMA content were exposed to laser beam. For these tests, a TruMark Station 100 laser-marking machine (Trumpf Group, Ditzingen, Germany), equipped with a 1064 nm Nd: YAG laser, was used. Using the TruTropsMark software (Trumpf Group, Ditzingen, Germany), graphic patterns with dimensions of 20 × 20 mm^2^ were applied to the surfaces of the test samples. In the research, a constant frequency of laser pulses, variable speed of the laser beam, and variable path width were used. Figure 2 shows images of graphic patterns resulting from the interference of the laser beam on the molding surfaces with differing roughness. A detailed list of laser-marking conditions is presented in Table 3.

### 2.4. Spectrophotometric Color Analysis

The differences in color between the marked surfaces were determined on the basis of colorimetric tests. The Ci62 sphere spectrophotometer (X-Rite, Grand Rapids, MI, USA) was used. The CIELab color space was applied to analyze the test results, within which the parameters L* (brightness, values from 0—black to 100—white), a* (color change from green to red), and b* (color change from yellow to blue) were determined for each measurement area. Color measurements were made on laser-marked pattern and on background of samples (unmarked surfaces). Five readouts were taken on each surface. Parameter ΔE* was adopted as the key for assessing the effectiveness of laser marking on the surfaces of PP samples with different roughness. This choice was due to the color difference and the contrast between the pattern symbol and the unmarked surface having the greatest influence on the effectiveness of laser marking. This parameter was calculated from the following formula:

(1) $${\;\Delta E}^{*}=\sqrt{{\left({\Delta L}^{*}\right)}^{2}+{\left({\Delta a}^{*}\right)}^{2}+{\left({\Delta b}^{*}\right)}^{2}}$$

### 2.5. Surface State Analysis

The surface state was defined in the individual areas of both molding inserts and the appropriate zones of the moldings. Measurements were also carried out on the surfaces of moldings after the laser-marking process. Their assessment was made by determining the roughness parameters using a VHX-7000 digital microscope (Keyence, Osaka, Japan), equipped with a universal VH0Z100R zoom lens with a possible magnification of 100–1000×. Selected parameters of 2D linear roughness and 3D surface topography were analyzed. The Ra (arithmetical mean deviation of the assessed profile) and Sa (arithmetical mean height of the surface) parameters for the surface state description of mold cavities and samples were used. Those parameters were calculated from the following formula:

(2) $$Ra=\frac{1}{\partial r}\int_{0}^{\partial r}\left|Z\left(x\right)\right|dx$$

(3) $$Sa=\frac{1}{A}\overset{\;}{\underset{A}{\iint }}\left|Z\left(x,y\right)\right|dxdy$$

The measurement of the linear roughness was carried out according to the ISO 4288 standard. Due to the roughness value, the length of the elementary section λc for the first mold insert (P, S, O) was 0.8 mm, while for the second (DA, DB, DC), it equaled 2.5 mm. On each of the surfaces, 10 measurements were made. The 3D surface roughness test was carried out by scanning of a selected fragment of the surface with dimensions of 3 × 3 mm, and then, using the “precise depth composition“ function, 100 individual photos were taken between the highest and the lowest point. Obtained images were combined and used for the analysis of the surface state. Additionally, the obtained results were compared to the VDI 3400 scale, which is commonly used to assess the surface state of mold cavities and samples. All measurements were carried out at a magnification of 400×. Sample images were also presented. This allowed assessing the influence of the additives content on the surface topography of the obtained pattern.

## 3. Results

### 3.1. Analysis of Surface State Parameters

The starting point for further analysis is the results of measurements of Ra and Sa surface parameters of the mold inserts and the obtained moldings (Table 4). The obtained results show that samples, after being taken out from the injection mold, do not map the roughness parameters of the molding cavity. The Ra and Sa values were higher for the surfaces of the PP samples than for the surfaces of molding inserts.

**Figure 3:**
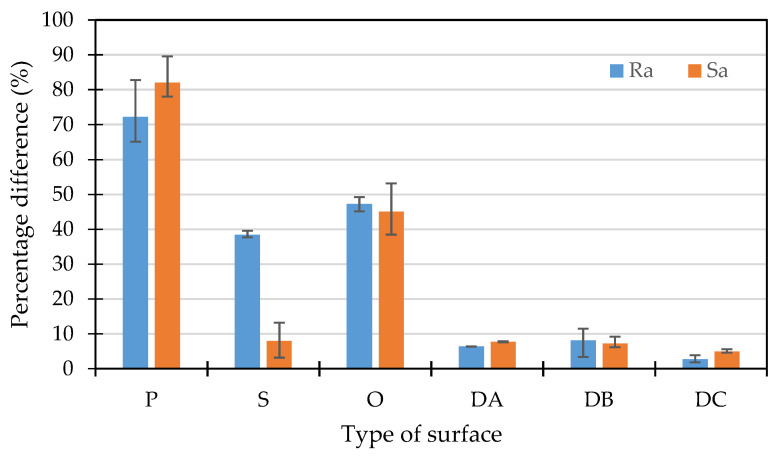
Percentage differences in the surface finish parameters (Ra, Sa) between the mold cavities and the obtained moldings.

The effect is due to the process parameters (melt and mold temperature, injection and holding pressure, injection speed) that influence melt viscosity and map the surface of the molding cavity. The results analysis in Figure 3 indicates that as the roughness of the cavity increases, the obtained moldings map the roughness of the mold cavity to a greater extent. It was found that the difference between the surface roughness of the obtained molding and the roughness of the part “P” of the insert is about 70% (“P” bars), while in the case of the surface after EDM process, the differences ranged from about 3% to 5% (“DC” bars).

The great difference between the polished and honed surfaces is due to the use of fine finishing. The difference between the Ra and Sa parameters for the grinding surface (“S” bars) comes from its directionality obtained in the technological process, which then affects the measurement method.

**Figure 4:**
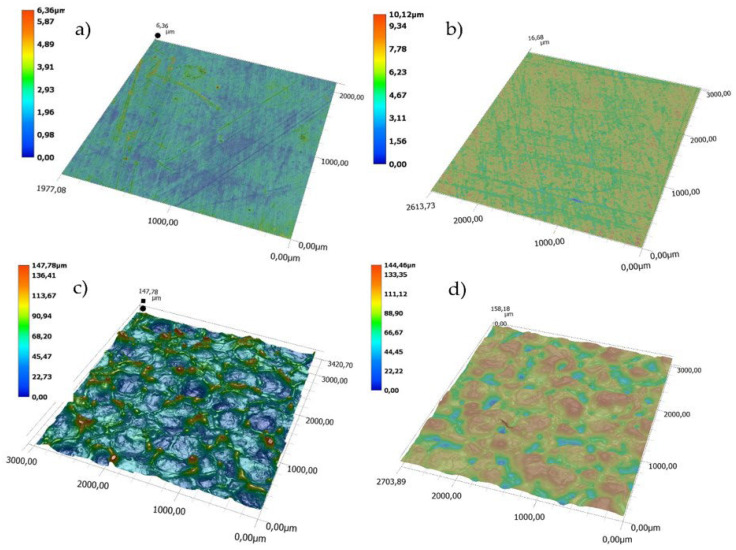
Topography: (**a**) polished surface (P) of the mold cavity, (**b**) polished surface (P) of the molding, (**c**) EDM surface (DC) of the mold cavity, (**d**) EDM surface (DC) of the molding.

A comparison of topographies of the polished (P) and the EDM (DC) surfaces is presented in Figure 4. For the polished surface, the topographic difference (between the lowest and the highest points on the 3D map) is approximate 6 µm for the mold cavity and approximately 10 µm for the moldings. In the case of the DC surfaces, the greatest topographic differences are 148 µm and 145 µm, respectively.

The laser beam on molding surfaces significantly influences its roughness at the point of marking. It was found that both the LMA content and the surface roughness before the marking process have a significant impact on the efficiency of the process. The results presented in the further part of the analysis refer to the marking using the parameters of the laser beam A (450 mm/s, 0.03 mm, 15 kHz). Table 5 shows the influence of the LMA content on the roughness parameters of the sample’s surface after the laser-marking process. For such parameters, the highest differences between the roughness of the graphic pattern and the background were obtained. The influence of other parameters on the surface roughness may be the subject of further studies.

The roughness parameters for surfaces with a high degree of finish (P, S, O) changed to the greatest extent. The value of the Ra parameter before the marking process for the polished surface was equal to 0.62, whereas after the marking process, the values were 0.82 (0 wt% LMA) and 5.49 (2.5 wt% LMA). This proves the purposefulness in using LMA to laser-mark PP moldings. These additives significantly increased the absorption of electromagnetic radiation energy. It was found that for the EDM molding insert, the influence of LMA content on Ra and Sa parameters is smaller than that of the degree of its finish.

**Figure 5:**
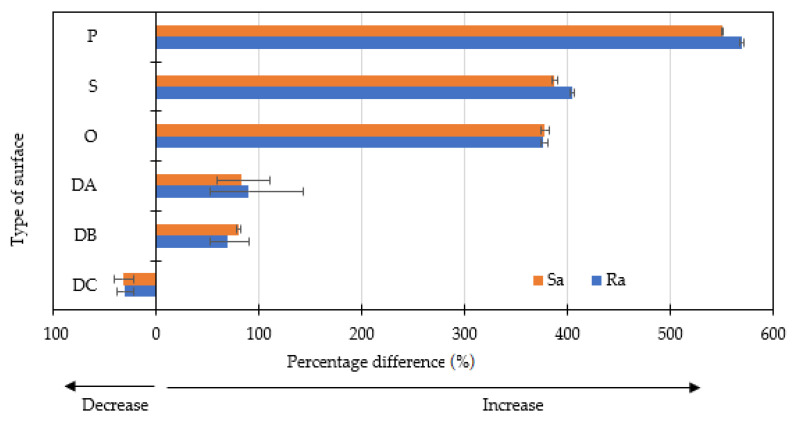
Differences in the surface state parameters (Ra, Sa) between the graphic pattern on the samples with 0 wt% LMA and 2.5 wt% LMA. Laser marking was carried out using the A parameters.

The percentage difference of Ra and Sa parameters for the graphic pattern on samples containing 0 wt% and 2.5 wt% LMA for the polished surface was nearly 600% (see Figure 5). The roughness of the graphic sign for grounded and honed surfaces for moldings containing 2.5 wt% LMA was higher than 400% compared to the same unfilled sample. This proves that the application of a marking additive is most effective for moldings with a high degree of surface finish. Much smaller differences were noted for samples with different LMA content obtained in EDM mold cavity. In the case of DA and DB surfaces, an increase in roughness for marked areas was observed on samples by 2.5 wt% LMA by about 80% compared to samples with 0 wt% LMA. Moreover, for the surface with the highest Ra and Sa parameters, DC, a decrease in the roughness of the pattern for the samples by 2.5 wt% LMA, compared to the unfilled samples, was noted. The high concentration of the modifier has resulted in a strong interference of the electromagnetic radiation beam on the surface, but in combination with the roughness increase, the intensity of this effect was reduced. Due to the strong concentration of the energy, a fragment of the surface layer was molted, and the thermal processes, such as foaming or carbonization, smoothed the rough surface. This proves that in some cases, the marking additive may lower the roughness relative to the unmarked sample. Additionally, it was noticed that for DA and DB surfaces after the marking process, both Ra and Sa values for samples with 0 wt% LMA were lower than those obtained for the unmarked surface. In the case of the DC surface with 0 wt% LMA content, the roughness of the graphic pattern was higher than that obtained for the unmarked surface. Appropriate dosing of the LMA may therefore result in a decrease or increase in roughness of pattern compared to the background surface.

**Figure 6:**
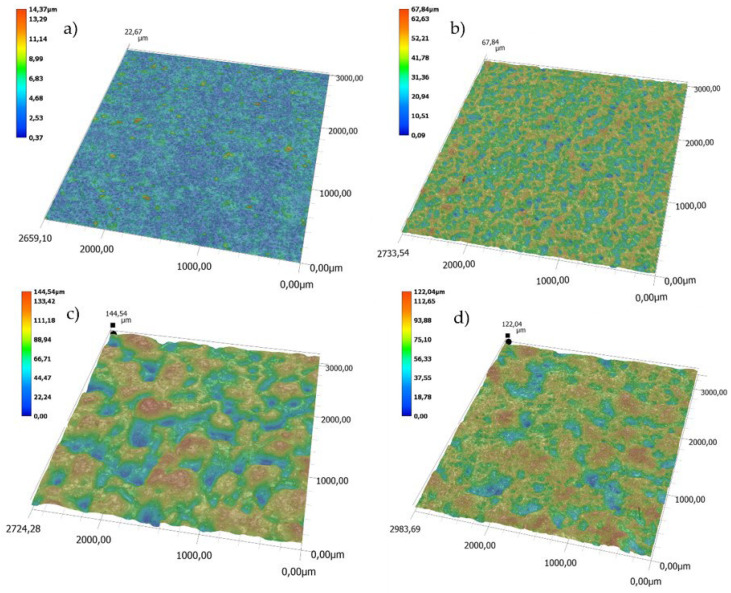
Influence of the LMA content on the surface topography of samples after the laser-marking process (laser A parameters): (**a**) surface P—0 wt% LMA; (**b**) surface P—2.5 wt% LMA; (**c**) surface DC—0 wt% LMA; (**d**) surface DC—2.5 wt% LMA.

Examples of the effects of laser energy on samples with different LMA content are shown on Figure 6. The LMA addition to the PP matrix caused a fourfold increase in the difference between the lowest and the highest points on 3D maps from about 14 µm to 68 µm for the polished surface (P). It can be observed that the surface layer of the tested sample was completely remelted. In the case of the “DC” molding, the impact of laser energy caused the surface to be partially melted and smoothed the tops of the pick profile (change in the difference in surface topography by approximately 18%).

### 3.2. Color Changes Analysis

**Figure 7:**
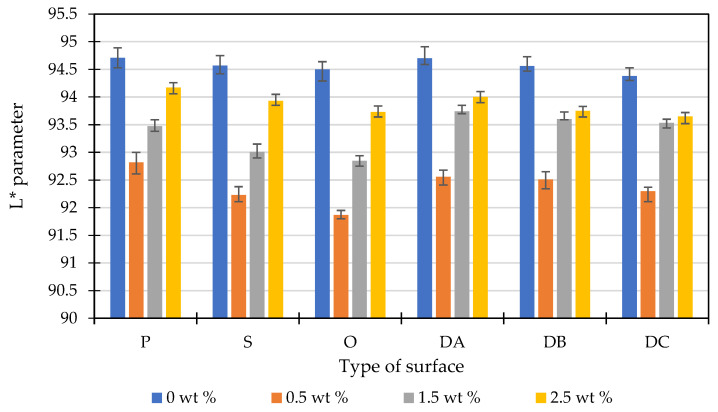
Effect of the content of the laser-marking additive on the brightness of the sample depending on the surface finishing.

Based on the colorimetric test, it was stated that the content of the marking additive influences the brightness of the polypropylene moldings (see Figure 7). It can be observed that the lowest values of the L* index were recorded for all types of surfaces with the LMA content equal to 0.5 wt%. These moldings were relatively darkest. The highest L* values were recorded for the unfilled samples. However, the overall differences in the L* parameter for all surfaces were insignificant. They ranged from 91.87 (0.5 wt% LMA—honed surface) to 94.71 (0 wt% LMA—polished surface). It was also found that the highest L* values for the polished samples, which belong to the high-finish surface molding group (sample: P, S, O) were obtained. For EDM group samples (DA, DB, DC), the highest values of the described parameter for the DA surface were obtained.

The filling of additives into the polypropylene matrix not only resulted in obtaining the white color of the moldings but also allowed the effectively application of graphic patterns on their surfaces. The occurrence of LMA in the PP matrix caused a significant increase in the contrast between the graphic pattern and the background (see Table 6). The color shade of the obtained graphic field depended on the laser operating parameters used and the LMA content. The change in the observed color of the marked surfaces mostly results from L* parameter (brightness), the values of which changed to the greatest extent, from about 44 (“DA” surface, A parameters of laser beam) to 90 (“P” surface, D parameters of laser beam). Values of CIELab parameters obtained for the graphic pattern compared to the background are presented in Table 6.

Moreover, the color differences were affected to a lesser extent by positive changes in the values of the parameter components on the “a” axis—from 0.29 for the P surface (0 wt% LMA; D parameters of laser beam) to 3.18 (DA surface—2.5 wt% LMA; A parameters of the laser beam) and in the range from 0.44 (DB surface; 0 wt% LMA; D parameters of the laser beam) to 6.51 (DA surface—2.5 wt% LMA; A parameters of the laser beam) on the “b” axis. The addition of LMA to the PP matrix increased values of both “a” (in the direction of increased red color saturation) and “b” (in the direction of increased yellow color saturation) parameters. As a result, taking all the described parameters into account, images of the laser-marked areas were characterized by an increased contrast between the graphic pattern and the background surface of the molded part. On samples with a higher LMA content and for A and B laser parameters (described in Table 3), graphic patterns in shades of black were obtained with clearly warm tones. The patterns made with C and D laser parameters were characterized by shades of gray for samples with a lower LMA content.

**Figure 8:**
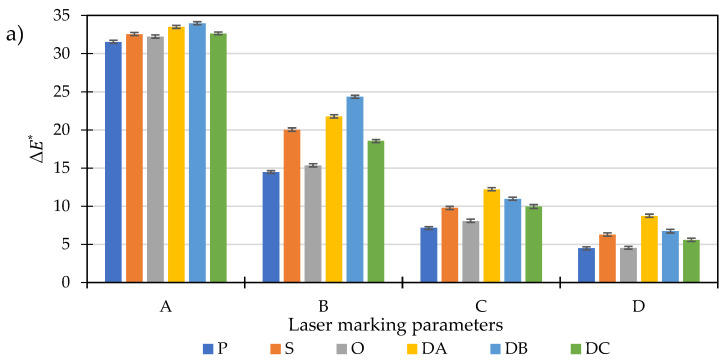

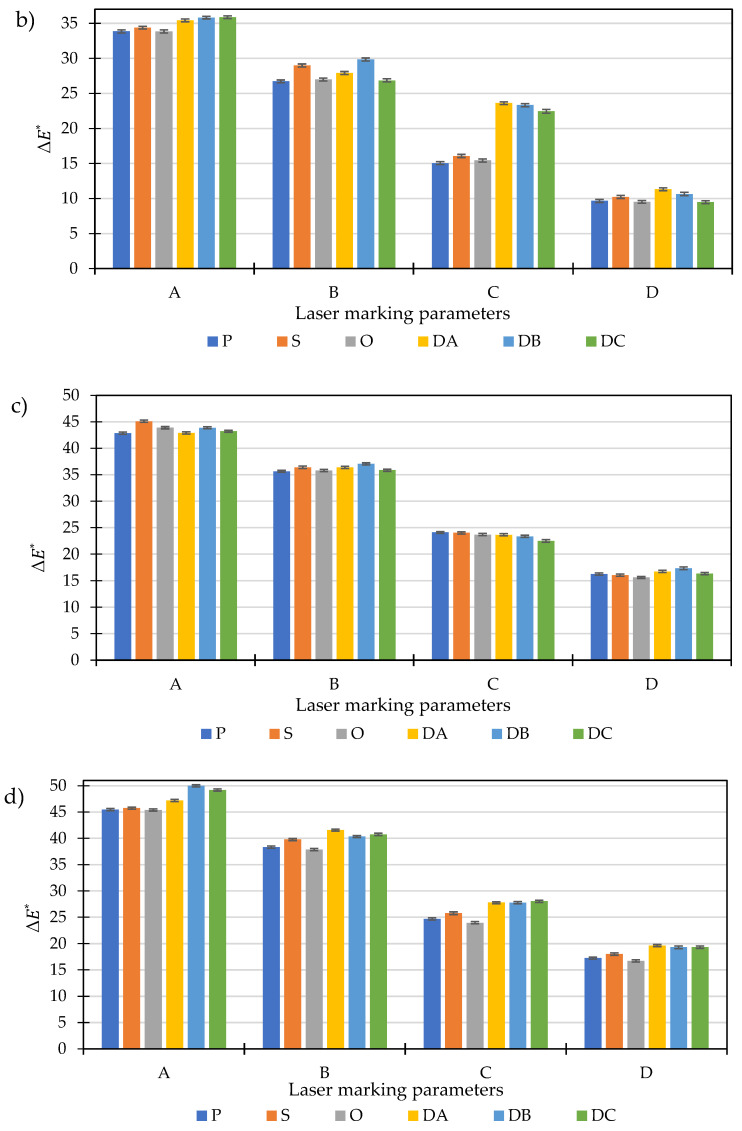
Effect of the content of the laser-energy-absorbing additive on the ΔE* parameter (total color deviation) in relation to the surface used and the laser parameters: (**a**) 0.0 wt% LMA additive content; (**b**) 0.5 wt% LMA additive; (**c**) 1.5 wt% additive; (**d**) 2.5 wt% LMA additive content.

For the unfilled PP samples, the density of the laser energy has the greatest impact on the change in ΔE* (Figure 8a). The higher the value of this parameter, the greater the observed changes on the molding surface. The use of laser-marking C parameters with a minimum LMA content of 0.5 wt% clearly shows the influence of the surface state on the changes in the ΔE* parameter (Figure 8b). The value of the ΔE* parameter is mainly determined by the L* parameter (Table 6). A further increase in LMA content to 1.5 wt% limits the influence of the surface state of white-colored polypropylene moldings on the quality of the obtained contrast between the graphic patterns (Figure 8c). Further increasing the LMA content to 2.5 wt% enhances the contrast only for the selected samples (Figure 8d).

For each analyzed surface, along with the increase in LMA content, the key assessment parameter ΔE* increased its value. The result is an increased contrast effect between the background and the marked surface. The changes in ΔE* for PP samples with a different surface finish ranged from about 4.49 (0 wt% LMA, polished surface, D laser parameters) to 49.97 (2.5 wt% LMA, DB surface, A laser parameters). The discrepancy in the results is therefore considerable. For samples with the same LMA content, the lowest contrast between the pattern and background was obtained for the polished and honed surfaces. Moreover, it was found that the obtained surfaces were characterized by the lowest roughness parameters Ra and Sa. Thus, it can be found that on surfaces with a high degree of finish, an adequate contrast between the graphic symbol and the background is more difficult to obtain. On the moldings with higher values of roughness parameters, the color difference was noticeable to a greater extent. This is related to the absorption coefficient of electromagnetic radiation by sample surfaces. The surface with higher roughness absorbs more energy due to the uneven reflection of the laser beam from its surface. Further analysis of the ΔE* value shows that the highest contrast between the graphic symbol and the background was obtained when using low laser head velocity (450 mm/s, 750 mm/s), combined with a high laser beam concentration (0.03 mm and 0.05 mm) and low frequency of laser pulses (15 kHz). The increase in the velocity of the laser beam and the width of the path resulted in lower contrast for each of the tested samples. The content of 2.5 wt% LMA increased the contrast by about 30% compared to the unfilled samples. The ΔE* value higher than 40 allows for concluding that the LMA content in the tested range and the A and B laser parameters favor to the most significant extent the susceptibility of the material to the absorption of laser radiation. Increasing the LMA concentration above 2.5 wt% may only slightly change the contrast between the graphic pattern and the background.

### 3.3. Laser Surface Modification

**Figure 9:**
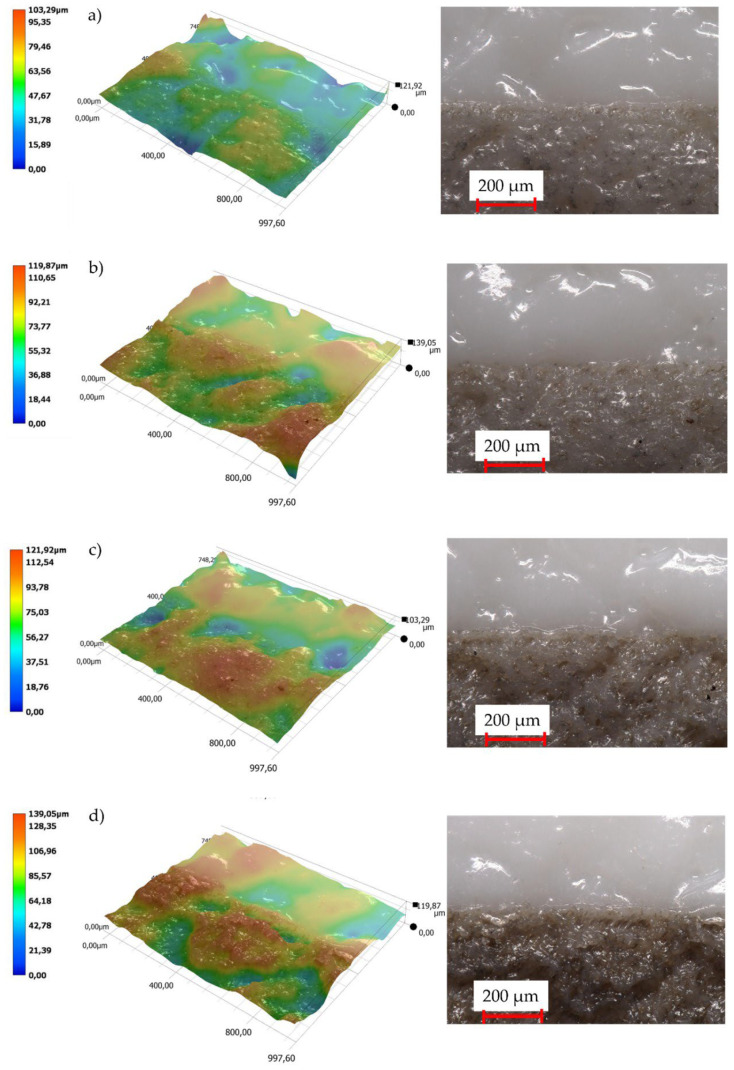
Influence of the LMA content on the surface topography (EDM DC) versus background (the upper half) after the marking process using laser A parameters: (**a**) 0.0 wt%, (**b**) 0.5 wt%, (**c**) 1.5 wt%, (**d**) 2.5 wt%.

Marking PP moldings containing the LMA resulted in modification of the skin layer, and the level of these changes depended on the content of the additive in the matrix (see Figure 9). It was found that one of the effects of the strong absorption of electromagnetic radiation by LMA particles is the foaming process on the surface of the molding. The formation of gas bubbles on the molding surface was observed due to local degradation of the skin layer (Figure 9b). The gas bubbles formed during the process were encapsulated under the thin top layer. Increasing the LMA content improved the absorption of laser radiation, the visible effect of which is an intense change in surface topography (Figure 9c,d).

**Figure 10:**
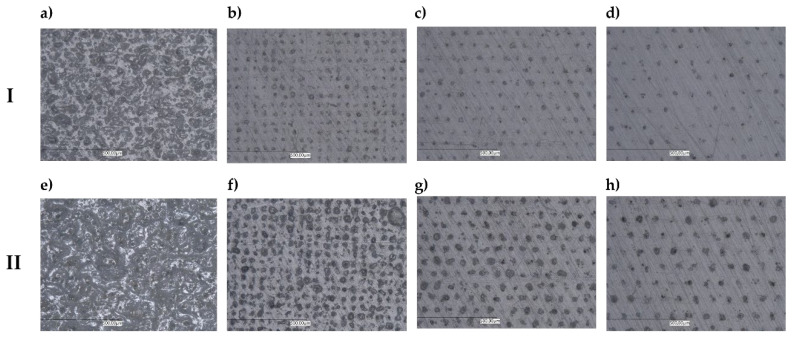
2D images of honed marked surfaces. Row I—0 wt% LMA. Row II—2.5 wt% LMA: (**a**,**e**) 450 mm/s, 0.03 mm; (**b**,**f**) 750 mm/s, 0.05 mm; (**c**,**g**) 1050 mm/s, 0.07 mm; (**d**,**h**) 1350 mm/s, 0.09 mm.

The use of low marking velocity (450 mm/s, 750 mm/s) and the frequency of 15 kHz resulted in multiple modifications of the marked surface through successive passes of the laser beam and overlapping of the laser beam interference points (Figure 10a,b,e,f). At higher frequencies and higher laser head speeds (1050 mm/s, 1350 mm/s), separated marked points on the surface were observed (Figure 10c,d,g,h). It was also observed that for moldings with higher LMA content (2.5 wt.%), the dimensions of the laser beam dots on the molding surface were larger than for samples with lower modifier content. This is due to the higher LMA content in the sample volume and confirms the important role of the modifier in achieving high marking efficiency for white-color samples. The observed change in the distance between the marked points as a result of increasing velocity and path width is the cause of the decrease in the value of the parameter ΔE* (compare Figure 8 and Figure 10). The observed irregularity in shapes of the marked dots may be caused by the uneven distribution of the LMA grains in the polypropylene matrix.

## 4. Conclusions

Conducted studies confirm the necessity of using LMA group modifiers for effective laser marking on the surfaces of white-colored PP moldings. Obtaining the appropriate contrast between the graphic pattern and the background depends on the concentration of the LMA. The research shows that the 0.5 wt% LMA content in the PP moldings resulted in obtaining a graphic pattern with high change in ΔE* (in the range from 30 to 35). Further increasing the LMA content to 2.5 wt% caused the most change in the ΔE* value for the A laser parameter (in the range from 45 to 50).

The highest contrast between the marked pattern and the background was noted on the surfaces of DB samples obtained from mold cavities produced by the EDM method (ΔE*—49.97). These values depended on the degree of surface finish of the samples. After marking, samples with higher surface roughness (EDM) contrasted significantly more with the white background when compared to samples with a high degree of surface finish (P, S, O). Based on these results, the surface condition of the molded parts can be related to the intensity of the foaming and carbonization process at the laser beam interaction zones. Mild surfaces of PP molding are characterized by a low value of the radiation absorption coefficient. The incident laser radiation is reflected to a greater extent than for samples with high roughness. Rough surfaces can absorb more beam energy, making the graphic pattern more visible. The laser energy changes the surface state parameters (Ra, Sa) by activating the LMA marking additive. Laser marking of white-colored polypropylene samples should be performed at a low velocity of the laser head (450 mm/s, 750 mm/s) and laser beam exposure with high concentration (0.03 mm, 0.05 mm), operating with a low pulse frequency (15 kHz). With such selected process parameters, the contrast between the graphic pattern and the background on the skin layer of molded parts is satisfactory and meets the expectations for effective and permanent marking of the surfaces of the white PP moldings.

This paper presented samples obtained in the injection molding process. This method is one of the two most important polymer material processing technologies that can be automated according to Industry 4.0 principles. Under the assumptions made, each molded part can be identified by its automatic laser labeling, such as a QR code. This makes segregation in the recycling process of used products much easier. An additional advantage of this solution is the ability to replace other known methods of applying graphic signs on the surfaces of molded parts, i.e., in-mold labeling, pad printing, hot stamping, and barcode sticking. The laser-marking method reduces the use of other materials to label products, which in turn reduces CO_2_ emissions and complies with Eco-Design principles.

## Figures and Tables

**Table 1:** Extrusion parameters used to prepare granulate.

Process Parameter	Value
Extrusion head temperature (°C)	200
Temperature of the plasticizing unit zones (°C)	I-135, II-180, III-200, IV-200
Screw rotation speed (rpm/min)	140

**Table 2:** Injection-molding parameters used to prepare test samples.

Process Parameters	Value
Feed zone temperature (°C)	200
Transition zone temperature (°C)	210
Metering zone temperature (°C)	230
Nozzle temperature (°C)	230
Injection mold temperature (°C)	20
Holding time (s)	6
Cooling time (s)	16
Injection time (s)	0.68
Injection pressure (MPa)	78.75
Holding pressure (MPa)	7.5

**Table 3:** Laser-marking parameters used to prepare samples.

Process Parameters	Value			
Mode of work	pulse			
Wavelength (nm)	1064			
Pulse frequency (kHz)	15			
Efficiency (%)	100			
Head velocity (mm/s)	450	750	1050	1350
Path width (mm)	0.03	0.05	0.07	0.09
Energy density (J/mm^2^)	0.37	0.13	0.07	0.04
Description of marked area	A	B	C	D

**Table 4:** Roughness parameters for each type of mold cavity surface finish and for obtained moldings. PP samples without laser-marking additives (LMA), mold temperature 20 °C.

Surface Type	Sign.	Surface of Cavity			Surface of Molding		
		Ra (μm)	Sa (μm)	VDI 3400	Ra (μm)	Sa (μm)	VDI 3400
Polishing	P	0.36 ± 0.07	0.39 ± 0.03	CH 12	0.62 ± 0.09	0.71 ± 0.04	CH 18
Grinding	S	0.52 ± 0.09	0.75 ± 0.03	CH 15	0.72 ± 0.12	0.81 ± 0.07	CH 18
Honing	O	0.55 ± 0.04	0.71 ± 0.07	CH 15	0.81 ± 0.07	1.03 ± 0.05	CH 19
	DA	2.51 ± 0.17	2.98 ± 0.08	CH 29	2.67 ± 0.18	3.21 ± 0.09	CH 29
EDM	DB	4.91 ± 0.41	5.84 ± 0.25	CH 34	6.12 ± 0.63	6.26 ± 0.21	CH 36
	DC	13.89 ± 1.77	14.94 ± 0.41	CH 44	14.27 ± 15.68	15.68 ± 0.48	CH 44

Ra-arithmetical mean deviation of the assessed profile, Sa-arithmetical mean height of the surface.

**Table 5:** Roughness surfaces parameters after laser-marking process for moldings.

Surface Type	Sign.	LMA Content					
		0 wt%			2.5 wt%		
		Ra (μm)	Sa (μm)	VDI 3400	Ra (μm)	Sa (μm)	VDI 3400
Polishing	P	0.82 ± 0.08	0.91 ± 0.05	CH 12	5.49 ± 0.47	5.92 ± 0.24	CH 35
Grinding	S	1.26 ± 0.17	1.34 ± 0.07	CH 15	6.35 ± 0.62	6.53 ± 0.31	CH 37
Honing	O	0.94 ± 0.16	0.99 ± 0.09	CH 15	4.48 ± 0.35	4.73 ± 0.18	CH 33
	DA	2.13 ± 0.21	2.27 ± 0.12	CH 27	4.04 ± 0.47	4.15 ± 0.13	CH 33
EDM	DB	5.72 ± 0.49	6.02 ± 0.24	CH 36	9.67 ± 0.94	10.83 ± 0.61	CH 41
	DC	15.67 ± 2.21	17.08 ± 0.87	CH 44	10.78 ± 1.21	11.56 ± 0.51	CH 42

**Table 6:** Influence of LMA content, laser parameters, and surface finish of moldings on color change of areas exposed to laser radiation.

wt (%)		ΔL*				Δa*				Δb*			
	Sign.	Laser Beam Parameters				Laser Beam Parameters				Laser Beam Parameters			
		A	B	C	D	A	B	C	D	A	B	C	D
0	P	31.35	14.29	7.01	4.34	1.52	0.93	0.49	0.29	1.91	1.44	0.93	0.71
	S	32.55	20.05	9.81	6.32	1.54	1.14	0.67	0.45	1.58	1.04	0.91	0.64
	O	32.51	15.58	8.32	4.81	1.44	0.97	0.58	0.36	1.37	1.32	1.01	0.66
	DA	33.22	21.51	11.96	8.5	1.57	1.2	0.74	0.54	1.85	1.18	0.81	0.62
	DB	33.99	24.39	11.01	6.79	1.44	1.18	0.71	0.44	1.09	0.6	0.66	0.44
	DC	32.88	18.82	10.21	5.83	1.54	1.05	0.66	0.36	1.72	1.02	0.9	0.72
2.5	P	44.77	37.78	24.07	16.64	2.34	1.76	1.49	1.14	3.72	0.87	1.64	1.43
	S	45.01	39.13	25.06	17.29	2.07	1.7	1.52	1.17	2.64	0.38	1.45	1.23
	O	44.97	37.47	23.49	16.25	1.93	1.72	1.47	1.16	1.36	0.43	1.31	1.31
	DA	46.77	41.61	27.79	19.61	3.18	2.07	1.77	1.45	6.51	2.16	2.13	2.03
	DB	49.74	40.35	27.69	19.17	2.56	1.62	1.66	1.38	4.52	0.9	1.82	2.19
	DC	48.97	40.57	27.82	19.05	2.01	1.74	1.71	1.38	1.92	0.88	1.92	1.93

## Data Availability

Not applicable.
